# Validity of Diaphragm Volume Measurements Using Three-Dimensional Computed Tomography

**DOI:** 10.14789/jmj.JMJ22-0006-OA

**Published:** 2022-09-09

**Authors:** ABULAITI ABUDUREZAKE, TERUMASA MORITA, TAKUYA MORI, ATSUSHI AMANO

**Affiliations:** 1 Department of Cardiovascular Surgery, Juntendo University Graduate School of Medicine, Tokyo, Japan; 1 Department of Cardiovascular Surgery, Juntendo University Graduate School of Medicine, Tokyo, Japan

**Keywords:** diaphragm, diaphragm volume, respiratory muscles, three-dimensional computed tomography, workstation

## Abstract

**Objectives:**

The aim of this study was to measure the diaphragm volume using three-dimensional computed tomography (3D-CT) and verify its validity.

**Design:**

This was a retrospective study of existing samples.

**Methods:**

Participants comprised five male patients, aged 65-70 years, who underwent preoperative chest CT (with a slice thickness of 0.5 mm) before coronary artery bypass surgery. The diaphragm was selectively extracted using a workstation to reconstruct a stereoscopic image, and the total muscle volume was measured. To confirm the accuracy and reproducibility of diaphragm muscle volume measurements on CT, all cases were measured three times by two observers, and intraclass correlation coefficients (ICCs) and interobserver correlations were determined.

**Results:**

Observers #1 and #2 reported an average diaphragm volume of 256.7±33 cm^3^ and 259.3±36 cm^3^, respectively. The ICC analyses yielded Cronbach's alphas of 0.992 and 0.981 from both observers, and the interobserver correlation was 0.991. The ICC of a single measurement and the average measurement was 0.984 (95% confidence interval: 0.998-0.884) and 0.992 (95% confidence interval: 0.999-0.939), respectively.

**Conclusions:**

To our knowledge, this study is the first to standardize the method for measuring the total diaphragm volume and examine the reproducibility and validity of the new method. The diaphragm could be selectively extracted and reconstructed. Measurement of the total diaphragm muscle volume using a workstation to reconstruct a stereoscopic image is feasible and highly reproducible. This technique can be reliably employed to evaluate diaphragm volume, thickness, and morphology.

## Introduction

The diaphragm is the main muscle involved in respiration, and functions during breathing^1)^. Understanding its anatomy is important for surgical treatment. Furthermore, an accurate evaluation of respiratory function during surgery performed under general anesthesia is an absolute condition for safely performing intraoperative and postoperative management and directly affects the surgical outcome, especially in thoracic surgery with thoracotomy^2, 3)^. Mechanical ventilation (MV) is necessary during surgery, as well as after surgery in the intensive care unit (ICU)^4-6)^. Multiple studies have suggested that MV has a significant effect on the respiratory system, especially the respiratory muscles^7, 8)^. In animal experiments, inactivity of the diaphragm on MV and passive ventilation lasting for more than 18 hours induced atrophy in the muscle fibers of the diaphragm^7, 9)^. This is also the case for the human diaphragm; MV for 18-69 hours resulted in significant atrophy of the human diaphragm muscle fibers^10)^. A clinical study that measured muscle thickness using echography found that the overall rate of decrease in the thickness of the patient's diaphragm averaged 6% per day of MV^11)^. Furthermore, in clinical practice, diaphragm thickness has been shown to decrease rapidly within a few days of MV in approximately 40% of patients^10, 12)^. Recently, diaphragm thickness measured during resting tidal breathing, in a cycle of spontaneous breathing, was shown to predict extubation success^13, 14)^. However, the rate of reintubation within 24-72 hours of planned extubation ranged from 2% to 25%, with medical, pediatric, and multidisciplinary ICU patients at the highest risk^15, 16)^.

To date, diaphragm volume evaluations, which are also commonly used in the context of research areas such as sarcopenia, physical development, and aging^17), 18)^, have only been determined using muscle thickness on echography. However, using echography alone to measure muscle thickness is inadequate^19)^; echography cannot be used to evaluate the overall morphology and volume of the diaphragm. By contrast, three-dimensional computed tomography (3D-CT) can clearly reveal the cross-sectional thickness and can also be used to evaluate morphology and volume. Sometimes, it is very difficult to differentiate the diaphragm from the liver and that is why some researchers report only the left diaphragm volume associated with the pulmonary function test result^20)^. To our knowledge, several studies used workstation software applications to evaluate diaphragm volume^20-22)^; however, until now, there has been no definitive standard for diaphragm volume measurements^23)^. Therefore, this study aimed to standardize the method of measuring diaphragm volume using 3D-CT with new criteria and to examine the validity of the new method.

## Materials and Methods

All procedures performed in studies involving human participants were in accordance with the ethical standards of the institutional and/or national research committee and with the 1964 Declaration of Helsinki and its later amendments or comparable ethical standards. The study design was approved by the institutional review board of Juntendo University School of Medicine (approval number: 20-342). For retrospective medical record surveys that handle only existing samples, instead of omitting informed consent, information on the implementation of research, including the purpose of the research, is posted by the Department of Cardiovascular Surgery on the Juntendo University website. We guaranteed the opportunity for the research subjects to refuse. The information disclosure document used was approved by the institutional review board of Juntendo University School of Medicine. Information that can identify the research subjects is not included. In addition, we did not use the samples of the research subjects for purposes other than research purposes.

### Research subjects

This was a retrospective study of existing samples. The participants comprised patients who were hospitalized or admitted to the Department of Cardiovascular Surgery at Juntendo University School of Medicine between June 2017 and January 2019. The selection criteria were as follows: patients underwent chest CT to evaluate the graft before coronary artery bypass grafting during hospitalization, and CT was performed with a 0.5-mm slice thickness (320 row multidetector Aquilion ONE ViSION Edition, Canon Medical Systems, Japan). Five patients (all male: age, 68.2±1.5 years; weight, 70.7±8.1 kg; height, 164.7±3.1 cm; BMI, 25.96±2.16 kg/m^2^; BSA, 1.77±0.10 m^2^) met the abovementioned selection criteria during the study period.

**Figure 1:**
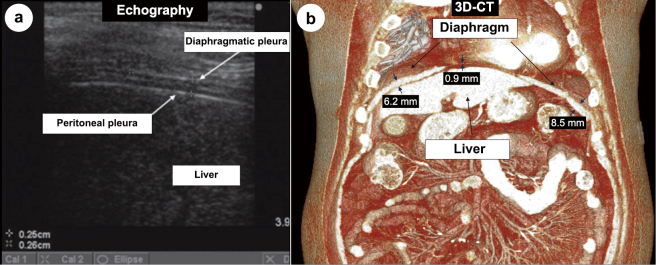
Echography and three-dimensional computed tomography (3D-CT). (a) The diaphragm, peritoneum, and liver^11)^ on echography. (b) The diaphragm and liver on 3D-CT. The diaphragm was selectively extracted from the CT data using a workstation to reconstruct a stereoscopic image, and the total muscle volume was measured. To confirm the accuracy and reproducibility of the diaphragm muscle volume measurements on CT, all cases were measured three times by two observers, and the intraclass correlation coefficients for each observer and interobserver correlations were determined.

As 3D-CT can evaluate, not only the difference in thickness but also the morphology and total volume, this method was used in the current study (Figure 1).

### 3D-CT assessment of the diaphragm

Diaphragm volume was measured based on the method used by previous researchers^24, 25)^. To evaluate the total volume of the diaphragm as much as possible, in this study, we standardized the method of measuring diaphragm volume using 3D-CT with a new standard and verified the validity of the new method. To confirm the accuracy and reproducibility of diaphragm muscle volume measurements on CT, measurements were recorded three times by two observers, and intraclass correlation coefficients (ICCs) as well as interobserver correlations were determined. The detailed explanation of this method is provided below.

**Figure 2:**
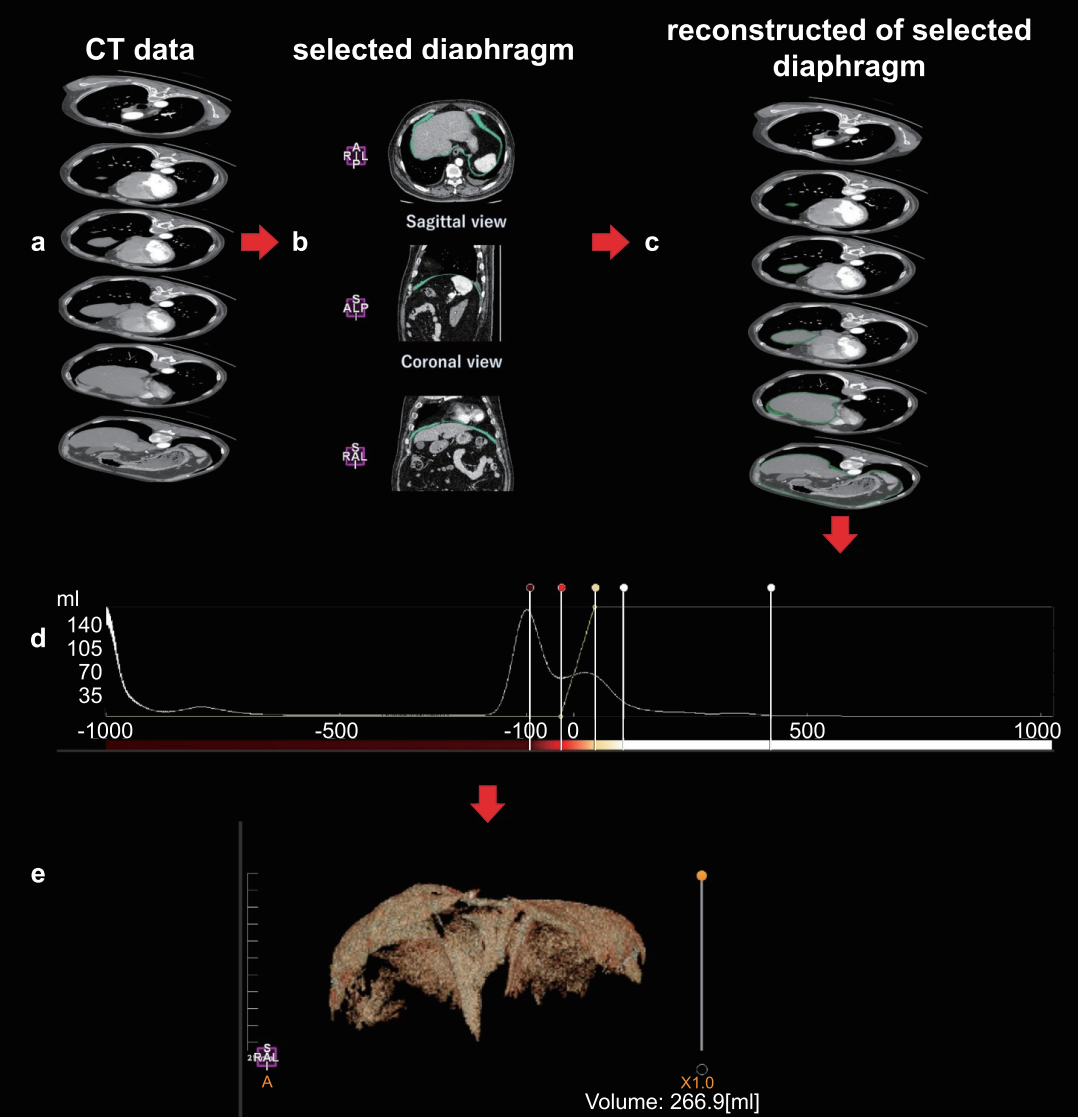
Operating steps for the assessment of the diaphragm volume during deep inspiration. (a) All computed tomography (CT) data are imported into the workstation software. (b) The diaphragm is selected and extracted. (c) The diaphragm is reconstructed. (d) The color mapping operation screen is shown. (e) The volume is measured.

All CT data were imported into the workstation software application, Attractive Medical Image Processor (PixSpace Co. Ltd., Japan) (Figure 2a). For each slice, the diaphragm was selected and extracted (Figure 2b). The diaphragm was then reconstructed using the contour trace function (Figure 2c). We performed color mapping to ensure that the entire diaphragm was visible (Figure 2-d), and the volume was measured using the 3D volumetry function (Figure 2e). Each of these steps is detailed below.

**Figure 3:**
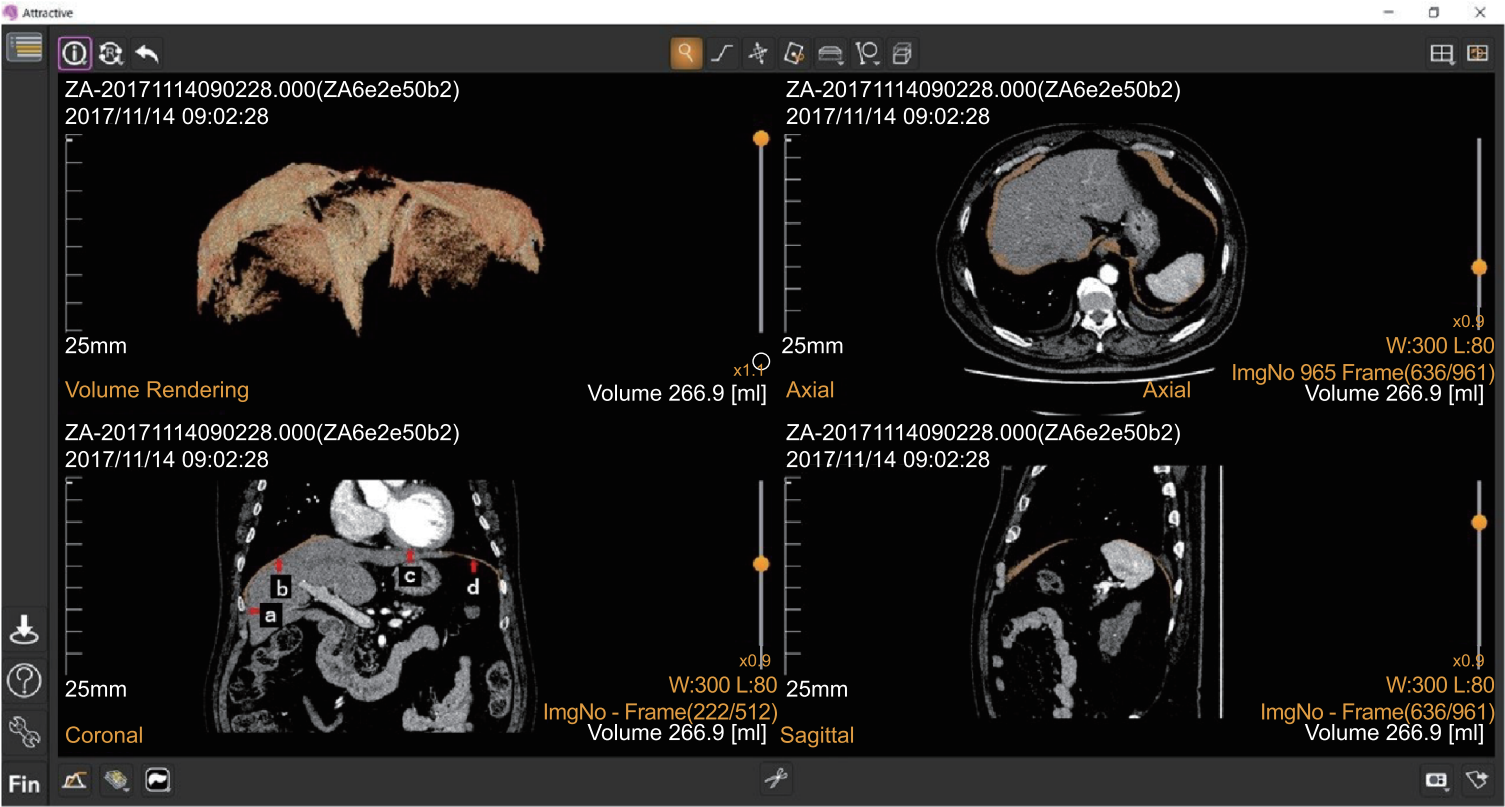
Diaphragm selection operation screen showing coronal, axial, and sagittal views of the diaphragm. (a) The location of the diaphragm attached to the chest wall. (b) Location of the diaphragm attached to the liver. (c) Location of the diaphragm attached to the heart. (d) Location of the diaphragm without attachment to any surrounding organs.

**Figure 4:**
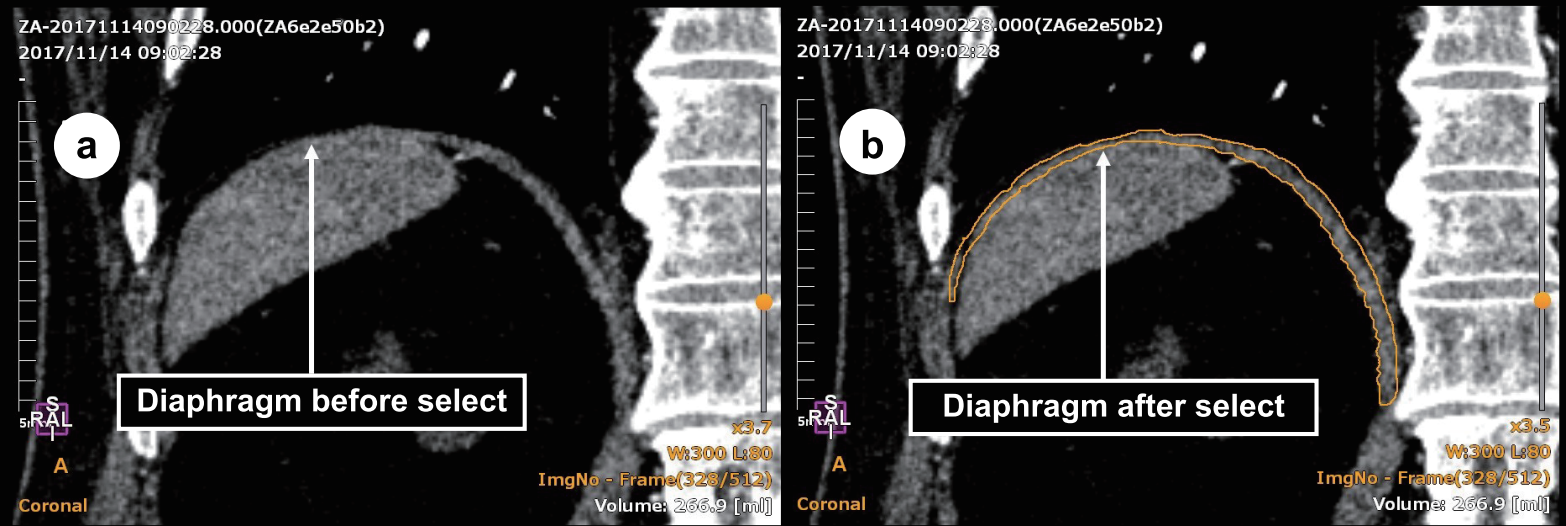
Diaphragm selection operation screen showing a coronal view of the diaphragm (a) before selection and (b) after selection. Diaphragm extraction: The diaphragm was separated from the heart, lungs, liver, and other surrounding tissues and then reconstructed (Figure 3 and Figure 4). Color mapping: Color mapping was performed such that the entire diaphragm was visible (Figure 2b).

For diaphragm selection, it was easy to select the diaphragm when there were no surrounding organs (Figure 3d). However, it was difficult to identify the diaphragm when surrounding organs were present, especially at locations in which the diaphragm and liver were attached (Figure 4). The following rules were used to standardize the selective extraction of the diaphragm: (1) The selection was first based on the coronal view and confirmed in the axial and sagittal views after extraction (Figure 3); (2) locations in which the diaphragm was attached to the chest wall and heart were not selected (Figure 3a and 3c); and (3) the zoom function was used more carefully to guide selection at the locations in which the diaphragm was attached to the liver and chest wall (Figure 3b and 4b).

For the removal of invisible areas, the 3D cutting function was used to remove air and fat tissue, which were selectively extracted from the diaphragm in invisible areas.

For the diaphragm volume measurement, in the final step, the diaphragm volume was measured using the 3D volumetry function (Figure 3), and observer #1 recorded three separate measurements for each case at AZE Virtual Place (AZE Co., Tokyo, Japan) using the same method. ICCs and interobserver correlations were then determined.

### Statistical analyses

SPSS software (version 25.0; SPSS Statistics, IBM Corp., Armonk, NY, USA) was used to perform the statistical analyses. All data are expressed as the mean ± standard deviation (SD) or 95% confidence intervals (CIs), and the significance level was set at less than 1%. The ICCs of each observer and between both observers were analyzed. The one-way random effects model with participant effects as random was applied to the ICCs of each observer, and a two-way random effects model with both participant effects and measure effects as random was applied to the ICCs of the interobserver differences. ICCs were interpreted following guidelines by Koo and Li (2016)^26)^ and classified as follows: below 0.50, poor; between 0.50 and 0.74, moderate; between 0.75 and 0.90, good; and above 0.90, excellent.

## Results

**Figure 5:**
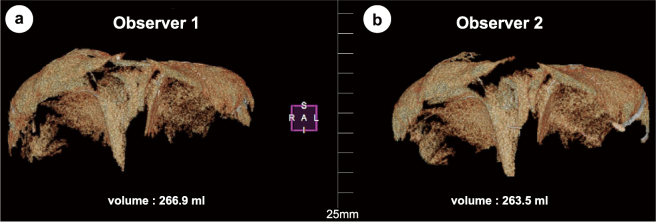
Diaphragm morphology and volume after reconstruction in case #4. The first measurement of (a) observer #1 and (b) observer #2 by workstation software PixSpace.

**Figure 6:**
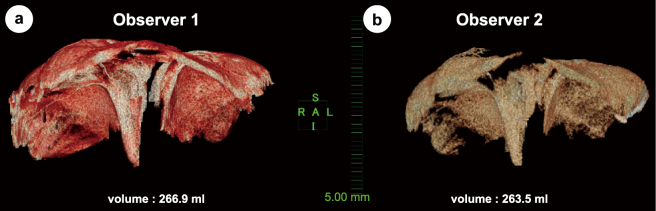
Diaphragm morphology and volume after reconstruction in case #4. The first measurements of (a) observer #1 by workstation software AZE and (b) observer #2 by workstation software PixSpace.

Age, weight, height, body mass index, and body surface area were normally distributed. In all cases, the diaphragm could be separated from the heart, lungs, liver, stomach, and other surrounding tissues, and the diaphragm volume could be evaluated (Figure 5 and 6).

Table 1 summarizes the detailed results and the average of all measurements by workstation software PixSpace according to the observer. The intraobserver agreement for the three measurements was excellent for observer #1, based on a Cronbach's alpha of 0.992, single-measurement ICC of 0.974 (95% CI: 0.997-0.892), and average-measurement ICC of 0.991 (95% CI: 0.999-0.961) (Table 2). The intraobserver agreement for the three measurements was also excellent in observer #2, based on a Cronbach's alpha of 0.981, single-measurement ICC of 0.955 (95% CI: 0.995-0.817), and average-measurement ICC of 0.984 (95% CI: 0.998-0.931) (Table 3). The interrater agreement, summarized in Table 4, showed a strong correlation among the measurements recorded by the two observers, with a Cronbach's alpha of 0.991, single-measurement ICC of 0.984 (95% CI: 0.998-0.884), and average-measurement ICC of 0.992 (95% CI: 0.999-0.939).

**Table 1:** Detailed results and average diaphragm volume across all measurements (first, second, and third) for observer #1 and observer #2 according to workstation software PixSpace

n=5	Observer #1				Observer #2			
	First (cm^3^)	Second (cm^3^)	Third (cm^3^)	Mean (cm^3^)	First (cm^3^)	Second (cm^3^)	Third (cm^3^)	Mean (cm^3^)
1	309.7	302.1	307.2	306.3	314.2	307.5	318.0	313.2
2	235.3	240.3	244.3	240.0	258.6	239.8	249.3	249.2
3	236.5	230.0	242.2	236.2	233.9	245.5	242.5	240.6
4	266.9	279.2	277.5	274.5	263.5	285.7	272.3	273.8
5	231.5	221.3	226.2	226.3	224.8	215.8	218.6	219.7
Mean	256.0	254.6	259.5	256.7	259.0	258.9	260.1	259.3
SD	33	35	33	33	35	37	38	36

Abbreviation: SD, standard deviation.

**Table 2:** Intraclass correlation coefficients for observer #1 by workstation software PixSpace

Measurement	ICC	95% CI		F-test with true value=0				Reliability statistics	
		Lower	Upper	Value	df1	df2	*P*-value	Cronbach's alpha	n
Single	0.974	0.892	0.997	115.413	4	10	<0.001	0.992	3
Average	0.991	0.961	0.999	115.413	4	10	<0.001		

A one-way random effects model was applied with participant effects as random. Abbreviations: CI, confidence interval; df, degrees of freedom; ICC, intraclass correlation coefficient.

**Table 3:** Intraclass correlation coefficients for observer #2 by workstation software PixSpace

Measurement	ICC	95% CI		F-test with true value=0				Reliability statistics	
		Lower	Upper	Value	df1	df2	*P*-value	Cronbach's alpha	n
Single	0.955	0.817	0.995	64.392	4	10	<0.001	0.981	3
Average	0.984	0.931	0.998	64.392	4	10	<0.001		

A one-way random effects model was applied with participant effects as random. Abbreviations: CI, confidence interval; df, degrees of freedom; ICC, intraclass correlation coefficient.

**Table 4:** Interrater agreement between measurements recorded by the two observers with workstation software PixSpace

Measurement	ICC^a^	95% CI		F-test with true value=0				Reliability statistics	
		Lower	Upper	Value	df1	df2	*P*-value	Cronbach's alpha	n
Single	0.984^b^	0.884	0.998	117.593	4	4	<0.001	0.991	2
Average	0.992	0.939	0.999	117.593	4	4	<0.001		

^a^ Type A ICCs using an absolute agreement definition. ^b^ The estimator was the same, regardless of whether the interaction effect was present. A two-way random effects model was applied with both participant effects and measure effects as random. Abbreviations: CI, confidence interval; df, degrees of freedom ICC, intraclass correlation coefficient.

Table 5 summarizes the detailed results and the average of all measurements by workstation software AZE to Observer #1. The intraobserver agreement for the three measurements by AZE was excellent for Observer #1, based on a Cronbach's alpha of 0.992, single-measurement ICC of 0.982 (95% CI: 0.998-0.922), and average-measurement ICC of 0.994 (95% CI: 0.999-0.972) (Table 6). The interrater agreement, summarized in Table 7, showed a high correlation between the measurements taken by the two observers between different workstation software, with a Cronbach's alpha of 0.984, single-measurement ICC of 0.956 (95% CI: 0.995-0.635), and average-measurement ICC of 0.978 (95% CI: 0.998-0.776).

**Table 5:** Detailed results and average diaphragm volume across all measurements (first, second, and third) for observer #1 by workstation software AZE

n=5	Observer #1(AZE)			
	First (cm^3^)	Second (cm^3^)	Third (cm^3^)	Mean (cm^3^)
1	309.6	308.4	301.7	306.6
2	238.3	235.3	238.9	237.5
3	227.7	222.6	229.5	226.6
4	258.6	272.8	260.0	263.8
5	230.5	224.2	227.7	227.5
Mean	252.9	252.7	251.6	252.4
SD	34	37	31	34

Abbreviation: SD, standard deviation.

**Table 6:** Intraclass correlation coefficients for observer #1 by workstation software AZE

Measurement	ICC	95% CI		F-test with true value=0				Reliability statistics	
		Lower	Upper	Value	df1	df2	*P*-value	Cronbach's alpha	n
Single	0.982	0.922	0.998	162.011	4	10	<0.001	0.992	3
Average	0.994	0.972	0.999	162.011	4	10	<0.001		

A one-way random effects model was applied with participant effects as random. Abbreviations: CI, confidence interval; df, degrees of freedom; ICC, intraclass correlation coefficient.

**Table 7:** Interrater agreement between measurements recorded by the two observers between different workstations

Measurement	ICC^a^	95% CI		F-test with true value=0				Reliability statistics	
		Lower	Upper	Value	df1	df2	*P*-value	Cronbach's alpha	n
Single	0.956^b^	0.635	0.995	63.902	4	4	<0.001	0.984	2
Average	0.978	0.776	0.998	63.902	4	4	<0.001		

^a^ Type A ICCs using an absolute agreement definition. ^b^ The estimator was the same, regardless of whether the interaction effect was present. A two-way random effects model was applied with both participant effects and measure effects as random. Abbreviations: CI, confidence interval; df, degrees of freedom ICC, intraclass correlation coefficient.

## Discussion

We performed an accurate evaluation of diaphragm morphology and volume using 3D-CT. Although, to our knowledge, this is not the first study to measure the total volume of the diaphragm^20-22)^, this study was the first to standardize the method for measuring the total diaphragm volume and examine the reproducibility and validity of the new method. Using 3D-CT, we were able to confirm the morphological evaluation of the entire diaphragm, including its form, thinness, and volume.

The diaphragm muscle volume reported in a previous study^21)^ was smaller than the mean diaphragm volume measured by our method. Although this could be due to differences in patient medical history and characteristics, as well as the software used, the difference in measurement method is also likely to be a major reason. Image analysis software generally use measurement methods that involve semi-automatic tracking and selection. This increases measurement speed, but the anatomy of the diaphragm makes it is difficult to perform complete selective extraction, due to the requirement for manual selection and data extraction. Thus, accurate measurement depends on the selection criteria as well as the operating ability of the measurer. Moreover, 1-mm slices were used in previous studies^21)^, whereas 0.5-mm slices were used in this study. We believe that manual selection and use of 0.5-mm slices enabled better evaluations; furthermore, in contrast to the previous study^21)^, we have described the operation procedure and selection criteria in detail to make measurement easier to reproduce. Fluctuations in intraclass and interrater correlations are likely to be due to differences in manually selected areas. We believe that this difference will be smaller if the technical training and operating procedures are standardized.

Although it remains uncertain whether the volume measured using the workstation Attractive Medical Image Processor software application, reflects the true volume of the diaphragm, other researchers have already confirmed that the volume of some organs measured on CT was the same as the actual volume. For example, in 1979, Heymsfield et al^27)^. were the first to accurately measure liver, kidney, and spleen volumes and masses using computerized axial tomography. The authors showed that this method was accurate by comparing the estimated and actual weights obtained at autopsy. The determination of liver volume on CT is now widely used in the field of liver surgery^28)^ and transplantation^29)^. In 2002, Surusuk et al^30)^. suggested that Heymsfield's method^27)^ is also reliable for the volumetric analysis of liver segments. Therefore, the diaphragm volume assessment in this study could have clinical value. However, this study was limited by its small sample size and retrospective nature. Consequently, the results were not sufficient to show the clinical usefulness of 3D-CT assessment of the diaphragm volume in comparison to that with a current reference imaging method, such as sonography.

In previous studies, conventional fluoroscopy, ultrasound, and magnetic resonance imaging (MRI) have been used to evaluate diaphragmatic functionality^31, 32)^. B- and M-mode ultrasound can easily diagnose diaphragmatic dysfunction and compare changes during the follow-up period^33)^, as well as provide real-time evaluation. It is often the imaging modality of choice and is a suitable bedside procedure. However, ultrasound is limited by operator dependency^33, 34)^ and cannot be used to evaluate the total diaphragm volume.

Several studies have shown that it is possible to evaluate diaphragm morphology and function using MRI^35, 36)^. MRI is a radiation-free technique that can provide static or dynamic evaluations, with the benefit of a wider field of view and more detailed soft tissue characterization^37)^. However, MRI, like ultrasound, cannot be used to evaluate the total diaphragm volume. Furthermore, the wide use of MRI is restricted by its limited availability, difficult scheduling, difficult patient preparation, and high costs^38)^. Because of its wider availability, CT scans are more commonly used as frontline imaging tests than MRI scans^36)^.

The present method also allows for the full use of existing CT data. The CT data used in this study were not initially collected to evaluate the diaphragm; rather, the CT data were obtained preoperatively. As existing CT data can be used, the problem of radiation exposure is reduced. As the conditions of the study subjects were limited, the postoperative diaphragm volume could not be measured in this study; however, changes in the diaphragm volume can be examined using existing CT data before and after surgery. Diaphragm volume measurements on 3D-CT can be expected to best combine the benefits of MRI and ultrasound and provide the most comprehensive assessment of the major inspiratory muscles.

In this study, the ICCs between the two observers using different workstation software were high for diaphragm volume measurements on 3D-CT. We considered the use of only echography to measure muscle thickness as insufficient for evaluating the diaphragm. Rather, we expect the present measurement method for evaluating the morphology and muscle volume of the diaphragm to be more useful when determining the timing of postoperative extubation. Diaphragmatic assessment is also commonly used in other areas of research such as in sarcopenia, physical development, and aging, where various situations and conditions can affect the ability of the diaphragm muscles to generate force. Although the diaphragm muscles can significantly weaken with age, leading to dysfunction, it is unclear whether there are any specific correlations between aging and the diaphragm.17) Total diaphragm volume evaluation by a new measurement method could be useful in clarifying any such relationships. Furthermore, in patients with a disease of the diaphragm, morphological abnormalities due to aging or disuse, and decreased muscle volume, aggressive respiratory rehabilitation is expected to improve diaphragm function, treatment results, and quality of life. In the future, this method could be used to evaluate respiratory function in surgical treatment and ICU patients. To avoid the issue of additional radiation exposure, CT data obtained during preoperative evaluations could be used.

This study has some limitations, including its small sample size (five subjects). However, it was possible to evaluate the diaphragm volume in all cases. Furthermore, the proposed measurement method involves radiation exposure, and it is still unknown whether the measured volume reflects the actual volume of the diaphragm. Additionally, technical standardization is necessary. Finally, these results may not be generalizable to other populations.

In conclusion, this study is the first to standardize the method for measuring the total diaphragm volume and examine the reproducibility and validity of the new method. The diaphragm could be selectively extracted and reconstructed. Measurement of the total diaphragm muscle volume using a workstation to reconstruct a stereoscopic image is feasible and highly reproducible. This technique can be reliably employed to evaluate diaphragm volume, thickness, and morphology.

## Availability of Data and Materials

All data generated or analyzed during this study are included in this article.

## Funding

The authors received no financial support for the research.

## Author contributions

All authors contributed to the conception of study design and data acquisition. AA, TM (TMorita), and TM (TMori) analyzed and interpreted the data and critically revised the manuscript for important intellectual content. AA was responsible for the investigation, methodology, drafting of the manuscript, validation, and visualization. TM (TMorita) was responsible for the project administration. AA (AAmano) critically revised the manuscript for important intellectual content and supervised the study. All authors read and approved the final manuscript.

## Conflicts of interest statement

The authors declare that there are no conflicts of interest.
